# Analysis of conjunctival fibroblasts from a proband with Schnyder corneal dystrophy

**Published:** 2008-07-09

**Authors:** Jodhbir S. Mehta, Eranga N. Vithana, Divya Venkataraman, Anandalakshmi Venkatraman, Rebekah Poh, Roger W. Beuerman, Tin Aung, Donald T.H. Tan

**Affiliations:** 1 Singapore Eye Research Institute, Singapore; 2 Singapore National Eye Centre, Singapore; 3 Department of Ophthalmology, Yong Loo Lin School of Medicine, National University of Singapore, Singapore

## Abstract

**Purpose:**

To analyze for the presence of lipids in conjunctival fibroblasts of a patient with Schnyder corneal dystrophy (SCD).

**Methods:**

A proband with SCD was identified, and the pedigree was examined. The proband underwent an automated lamellar therapeutic keratoplasty (ALTK). At the same time, the proband underwent a skin and conjunctival biopsy. Specimens were sent for histological and ultrastructural examination. Conjunctival fibroblasts were cultured from the biopsy specimen and stained with filipin.

**Results:**

The proband showed no evidence of recurrence following the ALTK procedure. Histopathological examination showed no evidence of hydrophobic lipids in the conjunctival or dermal fibroblasts. Lipid particles were detected in the cornea. Ultrastructural examination showed no lipid particles in the conjunctival fibroblasts. Cultured fibroblasts showed no evidence of intracellular unesterified cholesterol unless low density lipoprotein (LDL) was added to the culture medium.

**Conclusions:**

There was no evidence of lipid deposition in conjunctival or skin fibroblasts in our patient with SCD. The evidence suggests local factors are responsible for the lipid deposition in the cornea.

## Introduction

Schnyder corneal dystrophy (SCD; OMIM 121800) is a rare autosomal dominant inherited condition [1] that is characterized by bilateral anterior stromal haze or crystalline deposits in early life. Later, patients develop increasingly dense corneal arcus and diffuse stromal haze [1-8]. Crystalline deposition is not a prerequisite to diagnosing this condition, and 49% of patients have no evidence of crystalline deposits upon clinical examination [9]. The phenotypic presentation is caused by the abnormal deposition of cholesterol and phospholipids in the cornea [10]. Systemic hypercholesterolemia is associated with up to 66% of patients with SCD [11]. However, these abnormalities may occur without the presence of corneal abnormalities in pedigrees of SCD [12,13], suggesting an independent trait. Hence, it was hypothesized that local factors may affect lipid/cholesterol transport leading to deposition in the cornea. This was supported by the observations that early crystal deposition is often in the center of the cornea, implicating a temperature dependant enzyme defect [14,15] and that the cornea can actively uptake radioactive cholesterol [15].

In 1998, Battisti et al. [16] showed the presence of abnormal lipid storage in skin and cultured dermal fibroblasts from a pedigree with SCD. All the patients had normal serum lipid analysis. They concluded that the lipid storage abnormality was not limited to the cornea and that skin biopsy may be used to confirm the diagnosis. The aim of this study was to report on the analysis of conjunctival fibroblasts from a patient with SCD to examine for the presence of cholesterol accumulation.

## Methods

The protocol of the study adhered to the tenets of the Declaration of Helsinki and was approved by the institutional review board and ethics committees of the Singapore National Eye Centre and Singapore Eye Research Institute.

The proband and her family were evaluated clinically at the Singapore National Eye Centre by slit lamp biomicroscopy. Available clinical records, family history, and photographs were reviewed. Informed consent was obtained and the proband underwent an automated lamellar therapeutic keratoplasty (ALTK). A histopathologic study was performed on the excised corneal button and conjunctival (from two areas) and skin biopsies were taken during the same procedure.

**Figure 1:**
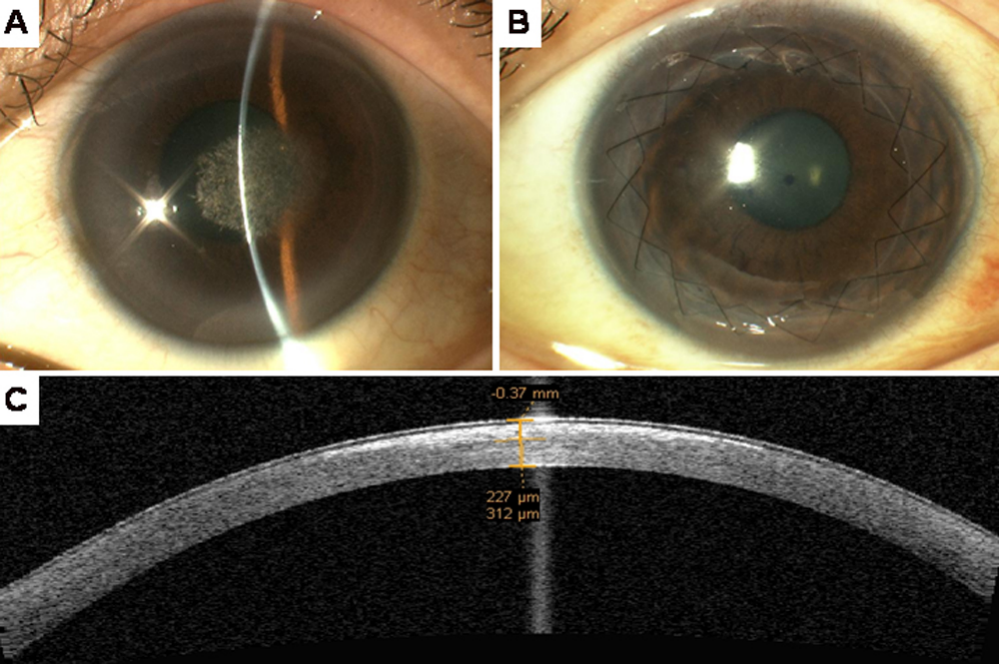
Color slit lamp photomicrographs of the proband IV:4. **A**: The image shows the proband’s left eye (OS) before ALTK. **B**: The image shows the same eye five years following ALTK with no signs of recurrence. **C**: Pre Pre-operative ASOCT of the proband showing hyper-reflective signal, indicating the depth of the crystalline lesions.

All members of the family of the pedigree underwent a complete ophthalmic examination. This included manifest refraction, visual acuity, contrast sensitivity with glare, corneal sensation, applanation tonometry, pupil reactions, motility, visual fields, corneal pachymetry, dilated fundus examination, and knee examination. Slit lamp examination and slit lamp photography was performed to assess and document the crystalline deposits and the degree of stromal haze and arcus lipoides to determine the disease status for each family member. Anterior segment optical coherence tomography (ASOCT, Carl Zeiss Meditec, Jena, Germany) was performed to assess depth of the lesions (Figure 1). All patients also underwent fasting serum blood cholesterol and triglyceride levels.

### Histopathology

The tissue specimens (conjunctiva, skin, and cornea) were paraffin sectioned and stained with hematoxylin and eosin and oil Red-O to detect for hydrophobic lipids (cholesteryl ester and triglyceride) by standard techniques.

### Cell culture

The conjunctival tissue was cut into 0.5 mm to 1 mm pieces and cultivated as explants on a sterile Petri dish with 500 µl of Dulbecco’s modified Eagle’s medium (Invitrogen, Carlsbad, CA) with nutrient mix F12 (DMEM Nut F12; Sigma Aldrich, St. Louis, MO) supplemented with 2 ng/ml human epidermal growth factor (Sigma Aldrich), 1 µg/ml bovine insulin (Sigma Aldrich), 0.5 µg/ml hydrocortisone (Sigma Aldrich), 10% heat-inactivated fetal bovine serum (Invitrogen), penicillin (5,000 units/ml), streptomycin (5,000 µg/ml; Sigma Aldrich), and fungizone (2.5 µg/ml; Sigma Aldrich). When cellular outgrowth from the explants was observed, the volume of the medium was increased to fully immerse the explants. The cells were incubated at 37 °C under 5% CO_2_/95% air with the medium changed every two to three days. Cultures were monitored under an inverted phase-contrast microscope (Axiovert; Carl Zeiss Meditec, Oberkochen, Germany). When a confluent layer of cells was obtained on the dish after 8–10 days, the cells were passaged. This was performed by detaching the cells with trypsin-EDTA (0.25%-1 mM; Invitrogen) for 2 min and then by seeding them on a six well plate containing DMEM Nutrient F12 medium supplemented as above. Following one passage, the cells were grown again to confluence in a six well plate. In half the six well plates, 5% low density lipoprotein (LDL; Sigma Aldrich) was added to act as a positive control. We then examined for non-esterified cholesterol using filipin stain (25µg/ml; a polyene antibiotic that selectively binds to the 3-beta-hydroxy-sterols; Sigma Aldrich) with a previously described method [17]. The filipin dye was excited with epi-illumination using ultraviolet light (Axioplan, Zeiss Light Microscope, Carl Zeiss), and fluorescence was viewed through a 510 nm barrier filter.

### Electron microscopy

The conjunctival tissue was removed and immediately fixed with cold 2% paraformaldehyde and 2% glutaraldehyde in 0.1 M sodium cacodylate buffer, pH 7.4 (Electron Microscopy Sciences, Hatfield, PA) at 4 °C overnight. The tissue was then washed in sodium cacodylate buffer and rinsed with distilled water. The tissue was then trimmed into smaller pieces. These samples were then post-fixed in 1% osmium tetroxide (Electron Microscopy Sciences). After extensive rinsing with distilled water, tissues were dehydrated in a graded series of ethanol and embedded in araldite (Electron Microscopy Sciences). All semi-thin sections (0.5–1 µm thick) were cut with Reichert-Jung Ultracut E Ultramicrotome (C. Reichert Optische Werke AG, Vienna, Austria), counter-stained with toluidine blue/basic fuchsin stain, and examined using a Zeiss Axioplan Light Microscope (Carl Zeiss). All ultra-thin sections of 60–80 nm were collected on copper grids, doubled-stained with uranyl acetate and lead citrate for 8 min each, and then viewed and photographed on a JEM 1220 electron microscope (JEOL, Tokyo, Japan) at 100 kV.

## Results

### Clinical findings

The proband (IV:4, Figure 2) was a 30-year-old Chinese woman who complained of increasing glare in both eyes. Uncorrected visual acuity (UCVA) was 20/80 OD and 20/80 OS. Best spectacle corrected vision (BSCVA) was 20/30 OD (−3.25/-1x165) and 20/ 30 OS (−3/-0.5x165). Pelli-Robson contrast sensitivity was OD 1.2, OS 1.5 with glare, OD 0.45, OS 0.6. Slit lamp examination of both eyes showed central axial subepithelial cholesterol crystals (Figure 1A) with arcus lipoides. There was no corneal vascularization or active inflammation. There was no stromal haze. Corneal sensation was normal in both eyes. She did not have genu valgum. Blood cholesterol was within normal limits. The depth of her lesions on ASOCT was 220 microns, and the central corneal thickness was 540 microns. She underwent a two stage automated lamellar therapeutic keratoplasty (ALTK; Moria, Antony, France) using a previously described technique [18]. Five years after the operation, the BSCVA was 20/20 (−0.75/-2.50x175) with no evidence of the crystals recurring (Figure 1B). Details of mutations in the *UBIAD1* gene in this pedigree (S171P) and clinical features of other members of this family have been previously described [19]. She is awaiting surgery on the other eye.

**Figure 2:**
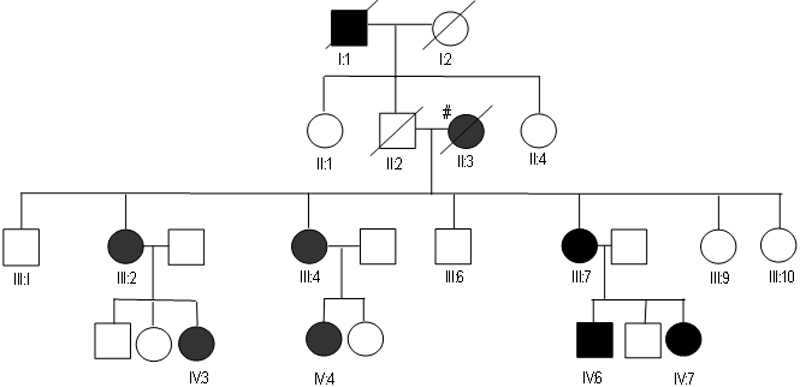
Pedigree of our Chinese family with Schnyder corneal dystrophy. The proband is IV:4. Eleven of the pedigree were examined, eight out of the nine affected members and three of the unaffected members. The sharp (hash mark) indicates that these members have hyperlipoproteinemia.

### Histopathology

**Figure 3:**
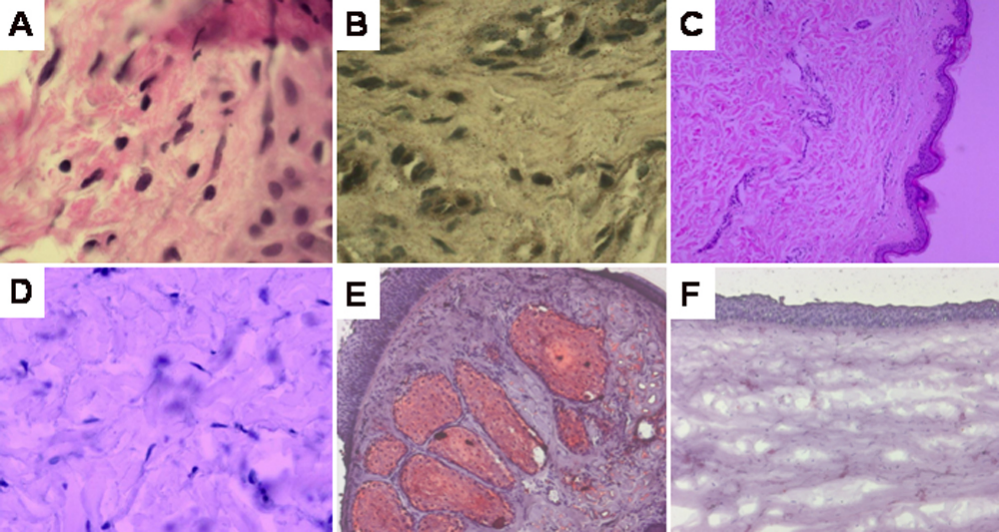
Histological sections of conjunctival and skin biopsy, from the proband. **A**: Hematoxylin and Eosin staining of conjunctival fibroblasts (40X). **B**: Negative result of oil Red-O staining of conjunctival fibroblasts (400X). **C**: Hematoxylin and Eosin staining of skin biopsy (40X). **D**: Negative result of oil Red-O staining of dermal fibroblasts (400X). **E**: Positive control for oil Red-O stain; lid margin staining the meiobium gland (100X). **F**: Negative control for oil Red-O stain; Normal cornea (100X).

Histopathologic evaluation of the excised corneal button revealed disruption and replacement of Bowman’s layer by connective tissue. There were fine spherical lipid particles deposited in the anterior part of the section to a level of 200 microns depth. Oil Red-O stained these particles, and there was also diffuse staining of the extracellular connective tissue matrix. Examination of the conjunctival and skin biopsy did not show the presence of triglyceride in the conjunctival fibroblasts or esterified cholesterol in the dermal fibroblasts as examined by oil Red-O staining (Figure 3A-D).

### Ultrastructural study of fibroblasts–electron microscopy

**Figure 4:**
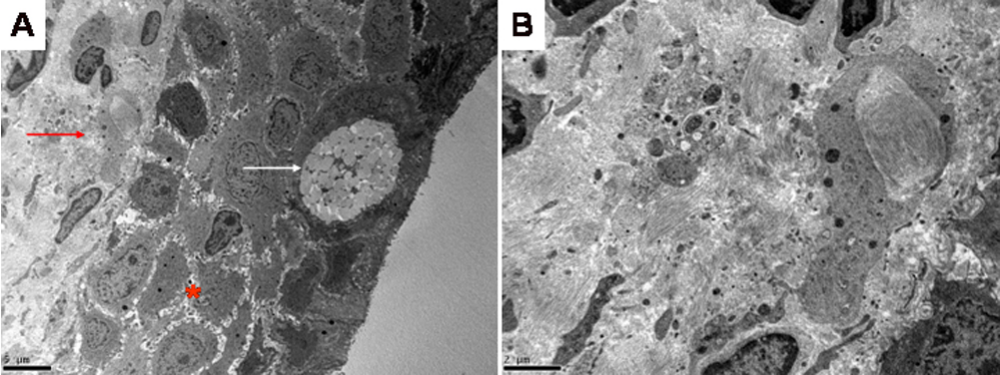
Transmission electron microscopy of conjunctival biopsy from the proband. **A**: Low power (2000X) section showing conjunctival epithelium (red star), goblet cell (white arrow) and conjunctival fibroblast (red arrow). **B**: High power section (5000X) of stromal fibroblastic tissue showing an absence of lipids in the cytoplasm of the conjunctival fibroblast.

Ultra-structural examination of the conjunctival biopsy did not show the presence of electron-lucent vacuoles in the fibroblast cytoplasm signifying lipid vacuoles (Figure 4).

### Cytochemical staining of unesterified cholesterol

**Figure 5:**
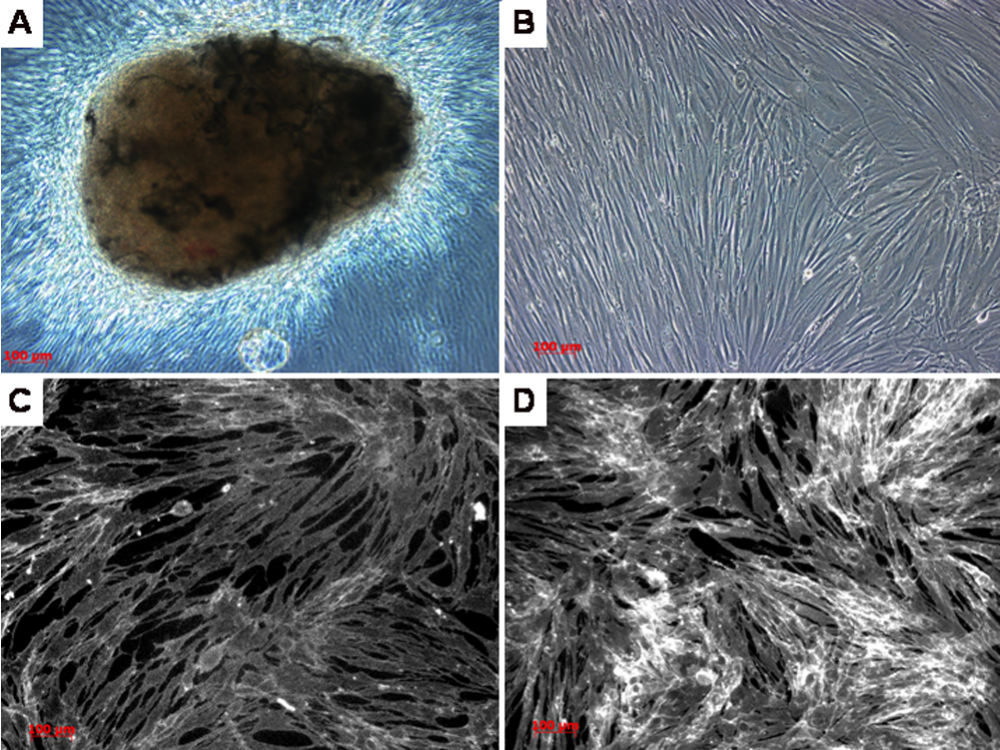
Fibroblast cell culture from the conjunctival biopsy of the proband. **A**: Fibroblastic cellular outgrowth from the conjunctival biopsy tissue. **B**: Following one week of cell culture, cellular confluence of fibroblasts is achieved. **C**: Following the addition of filipin stain, there is no evidence of cholesterol in the cultured conjunctival fibroblasts. **D**: Cells cultured with the addition of low density lipoprotein (LDL), acted as a positive control showing evidence of cholesterol with filipin staining. (Bar=100 microns).

Fluorescence microscope examination of the filipin-stained conjunctival fibroblasts from the wells without LDL did not show the presence of unesterified cholesterol in the cytoplasm (Figure 5C). The cells that had the addition of LDL showed cytoplasmic deposits of fluorescent inclusions throughout the cytoplasm especially in the perinuclear area (Figure 5D).

## Discussion

Schnyder corneal dystrophy is rare. The majority of reported cases have been from pedigrees of European ancestry [20]. More recently families from African American, Japan, and Turkish ethnicities have been described [21]. There are few reports in Chinese patients [22]. Recently, the gene for SCD has been identified [21,23], and the report by Battisti et al. [16] implies that the *UBIAD1* gene may play a role in lipid metabolism throughout the body. The authors showed the presence of lipid material in the skin fibroblasts from patients with SCD. Interestingly, all the patients had normal serum cholesterol levels, implying that perhaps the same local effect occurring in the eye may also be found in the skin. We hypothesized that if there was lipid deposition in dermal fibroblasts, then fibroblastic cells in other tissues may show lipid deposition. However, we were unable to show lipid deposition in our patient’s conjunctiva fibroblasts, and we could not verify the previously reported finding of lipids in the skin biopsy in SCD patients [16].

In the pedigree reported by Battisti et al. [16], two of the three examined patients had evidence of genu valgum. None of the members of our pedigree had this clinical sign. In a study of over 115 patients with SCD, this clinical sign was only documented in five [21]. The association between these two conditions is unclear. Some authors postulate an alteration in a pleiotropic gene, with variable penetrance [5]. Others suggest they are inherited as independent traits [24]. It may be that patients with genu valgum and SCD represent a more widespread syndromic disorder.

The identification of *UBIAD1* as the causative gene in SCD has shed more insight in the underlying pathogenesis of this condition. *UBIAD1* (previously know as *TERE 1* [transitional epithelia response protein 1] or *RP4–796F18*) is widely know to be expressed throughout human tissues [25]. *UBIAD1* interacts with the COOH-terminal portion of apo E [26], which is known to be involved in cholesterol transport, especially in mediating solubilization and in the removal of cholesterol from cells [27]. Hence, an obvious association between a defect in *UBIAD1* and cholesterol transportation is present [28,29]. However, *UBIAD1* also contains a prenyl-transferase domain, which is involved in cholesterol synthesis. Therefore, a defect in *UBIAD1* may be associated with an increase in local cholesterol synthesis [28]. The inability to detect lipid deposition in our dermal fibroblasts may be due to different mutations between our family and that described by Battisti et al. [16]. Although no correlation has been found between *UBIAD1* mutations and ocular phenotypes [21], there may be a genotype-phenotype correlation with extra-ocular deposition of lipids.

In summary, we were unable to identify the deposition of lipid in the skin and conjunctival fibroblasts of our proband with SCD. Larger samples are needed to verify our initial observations documented here, but the results suggest a localized abnormality in cholesterol metabolism in the cornea. The identification of *UBIAD1* offers new interest in understanding the underlying pathogenetic mechanism involved in this condition. Investigation into the tissue-specific expression of *UBIAD1* and research into the functional analysis of the effects of different mutations on the conformational changes in the protein may elucidate some of these questions.
